# Combining mental health and climate-smart agricultural interventions to improve food security in humanitarian settings: study protocol for the THRIVE cluster-randomized controlled trial with mothers in Nakivale refugee settlement, Uganda

**DOI:** 10.1186/s13063-025-09042-y

**Published:** 2025-09-01

**Authors:** Jonathan Hall, Herbert E. Ainamani, Phaidon T. B. Vassiliou, Stefan Döring, Gustaf Gredebäck, Kirsi Peltonen, Florian Scharpf, Umay Sen, Matthias Sutter, James I. Walsh, Tobias Hecker

**Affiliations:** 1 Department of Peace and Conflict Research, Uppsala University, Uppsala, Sweden; 2 Department of Mental Health, Kabale University School of Medicine, Kabale University, Kabale, Uganda; 3 Peace Research Institute Oslo (PRIO), Oslo, Norway; 4 Swedish Centre for Impacts of Climate Extremes (Climes), Uppsala University, Uppsala, Sweden; 5 Department of Psychology, Uppsala University, Uppsala, Sweden; 6 INVEST Research Flagship Centre, University of Turku, Turku, Finland; 7 Department of Psychology, Bielefeld University, Bielefeld, Germany; 8 Institute for Interdisciplinary Conflict and Violence Research, Bielefeld University, Bielefeld, Germany; 9 Max Planck Institute for Research on Collective Goods, Bonn, Germany; 10 Department of Economics, University of Cologne, Cologne, Germany; 11 Department of Public Finance, University of Innsbruck, Innsbruck, Austria; 12 Department of Political Science, School of Data Science, and Program in Public Policy, University of North Carolina at Charlotte, Charlotte, USA

**Keywords:** Adaptation, Agriculture, Armed conflict, Climate change, Food security, Home gardening, Humanitarian emergencies, Malnutrition, Mental health, Refugees, Self-help plus, Trial protocol, Uganda

## Abstract

**Background:**

Climate extremes in Africa threaten the food security of war-affected refugees, who often experience mental health challenges that hinder their capacity for agricultural adaptation. Cost-effective, climate-smart farming interventions are crucial for addressing food insecurity in humanitarian contexts, yet evidence on their effectiveness is limited, and the potential benefits of integrating them with mental health interventions remain unexplored. We hypothesize that the success of agricultural interventions, especially under adversity, is influenced by mental health and psychological functioning.

**Methods:**

This study employs a three-arm, parallel-group, cluster-randomized controlled trial (cRCT) in the Nakivale refugee settlement, Uganda. Thirty villages within the settlement will be randomized in a 1:1:1 allocation ratio to one of three conditions: Enhanced Usual Care, a Home Gardening Intervention (HGI) or HGI combined with the peer-delivered psychosocial intervention Self-Help Plus (SH + HGI). A total of 900 refugee mothers and their children (aged 3–4 years) will be enrolled, with 30 dyads per village. The primary outcome is food insecurity at 12 months post-intervention, assessed using the Food Insecurity Experience Scale (FIES). Secondary outcomes include dietary diversity, child malnutrition and mothers’ psychological distress. Data will be collected at baseline, 3-month and 12-month follow-ups. Primary analyses will use an intention-to-treat (ITT) approach.

**Discussion:**

This study will shed light on the role of mental health in agricultural adaptation for food security, evaluating the efficacy of scalable, cost-effective interventions in a refugee setting. The findings will have implications for the design and implementation of integrated food security and mental health programs in humanitarian and other resource-constrained settings.

**Trial registration:**

ClinicalTrials.gov NCT06425523. Registered on 24 May 2024.

**Supplementary Information:**

The online version contains supplementary material available at 10.1186/s13063-025-09042-y.

## Administrative information

Note: the numbers in curly brackets in this protocol refer to SPIRIT checklist item numbers. The order of the items has been modified to group similar items (see http://www.equator-network.org/reporting-guidelines/spirit-2013-statement-defining-standard-protocol-items-for-clinical-trials/).

**Table:** 

Title {1}	Combining mental health and climate-smart agricultural interventions to improve food security in humanitarian settings: Study Protocol for the THRIVE Cluster-Randomized Controlled Trial with Mothers in Nakivale Refugee Settlement, Uganda
Trial registration {2a and 2b}	ClinicalTrials.gov identifier: NCT06425523.
Protocol version {3}	Initial release at ClinicalTrials.gov: May 17, 2024. Last update at ClinicalTrials.gov: January 22, 2025. Version 3 of January 22, 2025.
Funding {4}	This work is supported by FORMAS (grant no. 2022–01573).
Author details {5a}	Jonathan Hall: Department of Peace and Conflict Research, Uppsala University, Uppsala, Sweden; Herbert E. Ainamani: Department of Mental Health, Kabale University School of Medicine, Kabale University, Kabale, Uganda; Phaidon T. B. Vassiliou: Department of Peace and Conflict Research, Uppsala University, Uppsala, Sweden; Stefan Döring: Department of Peace and Conflict Research, Uppsala University, Uppsala, Sweden; Peace Research Institute Oslo (PRIO), Oslo, Norway; Centre for Impacts of Climate Extremes (climes), Uppsala University, Uppsala, Sweden; Gustaf Gredebäck: Department of Psychology, Uppsala University, Uppsala, Sweden; Kirsi Peltonen: INVEST Research Flagship Centre, University of Turku, Turku, Finland; Florian Scharpf: Department of Psychology, Bielefeld University, Bielefeld, Germany; Institute for Interdisciplinary Conflict and Violence Research, Bielefeld University, Bielefeld, Germany; Umay Sen: Department of Psychology, Uppsala University, Uppsala, Sweden; Matthias Sutter: Max Planck Institute for Research on Collective Goods, Bonn, Germany; Department of Economics, University of Cologne, Cologne, Germany; Department of Public Finance, University of Innsbruck, Innsbruck, Austria; James I. Walsh: Department of Political Science, School of Data Science, and Program in Public Policy, University of North Carolina at Charlotte, Charlotte, USA; Tobias Hecker: Department of Psychology, Bielefeld University, Bielefeld, Germany; Institute for Interdisciplinary Conflict and Violence Research, Bielefeld University, Bielefeld, Germany
Name and contact information for the trial sponsor {5b}	This study is sponsored by Uppsala University. UCR-Uppsala Clinical Research center info@ucr.uu.se Uppsala Science Park Dag Hammarskjölds väg 38 751 85 Uppsala (SE)
Role of sponsor {5c}	The sponsor and funder have had no role in the design or submission of this protocol. They will have no role in the collection, management, analysis, and interpretation of data.

## Introduction

### Background and rationale {6a}

Climate change poses significant challenges across Africa, particularly for populations dependent on rain-fed agriculture. These communities face increasingly frequent and severe climate-related shocks, such as droughts and floods [1–3], which threaten food security and negatively impact both physical and mental health [4–6]. The situation is further complicated in regions affected by conflict and displacement, where fragile food systems and limited resources exacerbate vulnerabilities [7, 8]. Refugee settlements, in particular, are highly susceptible to climate extremes and their inhabitants face significant challenges in establishing self-reliant livelihoods [9, 10]. Furthermore, exposure to armed conflict and displacement increase mental health problems, which can hinder adaptive capacities [11, 12]. These interconnected issues create a cycle of adversity, resource scarcity, poor mental health and impaired adaptation [13–15].

Rigorous evaluations of interventions aimed at enhancing refugee resilience are crucial for sustainable development amidst climate change [3]. However, there is a lack of systematic research on effective strategies to integrate mental health support with climate-smart interventions, especially among vulnerable populations [16–18]. While the international community acknowledges climate-related malnutrition and invests in agricultural interventions [19, 20], and there is increasing awareness of the mental health gap in low- and middle-income countries [21–23], empirical evaluations of agricultural interventions in humanitarian crises remain limited [24, 25], and the role of mental health in agricultural adaptation is largely unexamined [26, 27].

We contend that successful agricultural adaptation to climate change relies not only on material resources and technical knowledge, but also on individuals’ psychological capacities to implement and maintain these practices [28, 29]. Refugees possess essential psychological skills for sustainable livelihoods, including the ability to learn, adapt, commit to long-term goals and engage in collaborative efforts [30, 31]. However, these skills are often compromised by mental health problems stemming from prolonged exposure to stress and adversity. This can lead to a state of allostatic overload, which disrupts cognitive functions and alters behaviors crucial for effective adaptation [32–35]. Therefore, we posit that addressing mental health will improve psychological functioning, thereby enhancing adaptive capacity [36–38].

To test this, this study aims to evaluate the impact of a climate-resilient, sustainable and scalable Home Gardening Intervention (HGI) on food security among war refugees in Nakivale refugee settlement, Uganda. Simultaneously, the study will evaluate whether enhancing participants’ mental health using the safe, cost-effective and validated psychosocial intervention Self-Help Plus (SH +) [39] can amplify the expected positive impact of HGI on food security.

## Objectives {7}

Our trial aims to test two main hypotheses:

H1: Participants in the villages allocated to the stand-alone Home Gardening Intervention will experience significantly improved food security at the 12-month endpoint, compared to participants in the control group.

H2: Participants receiving SH + in combination with HGI will exhibit superior food security outcomes compared to those receiving HGI alone.

## Trial design {8}

The 30 participating villages within the Nakivale refugee settlement will be the unit of randomization and will be assigned to one of three arms: (1) Enhanced Usual Care (EUC), (2) Home Gardening Intervention (HGI) or (3) a combined Self-Help Plus and Home Gardening Intervention (SH + HGI). To account for baseline differences associated with settlement patterns of recent arrivals, the randomization will be stratified and staggered, with villages allocated in three phases (see section on Participant timeline {13} for details). The trial is designed to demonstrate the superiority of HGI, and particularly SH + HGI, compared to EUC in improving food security outcomes at 12 months post-intervention. The cluster-randomized controlled trial (cRCT) design is essential due to the nature of the interventions, which are delivered at the community level, and to minimize the risk of contamination between the intervention groups that could arise from individual randomization within the same village.

## Methods: participants, interventions and outcomes

### Study setting {9}

This study will take place within the Nakivale refugee settlement, located in southwestern Uganda, Isingiro District. Nakivale is the second-largest refugee camp in Uganda, hosting over 216,000 individuals spread across 79 villages [40, 41]. The inhabitants face significant challenges due to extreme weather patterns, including heatwaves and arid land during dry periods, as well as devastating floods that damage homes and agricultural production during the rainy season [9]. The camp also grapples with numerous agricultural obstacles, including a lack of livestock and restricted access to diverse crops and agricultural training [42]. Food insecurity and mental health issues are major concerns, with high rates of malnutrition among women and children, limited dietary diversity, a strong dependence on external food aid [42] and a substantial proportion of the population suffering from conditions such as generalized anxiety disorders, posttraumatic stress disorder and major depressive disorder [43]. These conditions make Nakivale a particularly relevant setting to study the interplay between climate resilience, food security and mental health.

### Eligibility criteria {10}

The study will be conducted in 30 villages within the Nakivale refugee settlement. Villages were selected based on sufficient access to water for manual irrigation and land for agriculture. Highly urbanized villages and those currently participating in similar programs were excluded. Refugee mothers residing in these selected villages are eligible to participate if they meet the following inclusion criteria: having a child aged 3–4 years, having access to a plot of land suitable for farming, being able to speak and understand Kiswahili, being 18 years of age or older and not planning to move from the settlement within the next 12 months (the study duration). Furthermore, mothers must exhibit at least moderate levels of psychological distress to be included, defined as a score of five or higher on the Kessler 6 (K6) psychological distress scale [44].

Mothers will be excluded if they present an imminent risk of suicide, as assessed through a structured protocol incorporating the suicidality subscale of the Mini International Neuropsychiatric Interview [45]. Additional exclusion criteria include observable signs of psychosis, manic behaviors or intellectual disability that would impede the ability to understand and follow intervention instructions or participate in group sessions. Participants will also be excluded if the mothers express knowledge of their child’s cognitive or developmental impairment, or if the child does not live with the parent who is eligible to take part in the intervention. In the event that a prospective participant has multiple children within the age range of 3–4 years, the index child will be selected by asking the mother which child had their birthday most recently. Both mother and child must meet the eligibility criteria, and provide consent/assent, to be included.

Twenty model farmers will be selected based on their agricultural experience, standing in the community and willingness to participate in training and to provide ongoing support to participants. Self-Help Plus (SH +) facilitators will be selected based on prior working experience in the refugee settlement, social skills, proficiency in both Kiswahili and English and a minimum of secondary education.

### Who will take informed consent? {26a}

Informed consent will be obtained from all participating mothers by trained local enumerators, supervised by the on-site principal researcher. The enumerators will undergo thorough training on the screening, consent procedures and ethical considerations involved in the study. The enumerators will present an informed consent form to all eligible participants, explaining the purpose of the study, the procedures involved, the potential risks and benefits of participation, data confidentiality and the participants’ right to withdraw from the study at any time without penalty. Mothers will be asked to provide parental consent for their child’s inclusion in the study, and the children will be asked to assent. Literate participants will provide their consent by signing the informed consent form. For participants who are illiterate, a thumbprint will be used as a valid form of consent, with a witness present during the consent process.

### Additional consent provisions for collection and use of participant data and biological specimens {26b}

No biological specimens will be collected in this trial.

## Interventions

### Explanation for the choice of comparators {6b}

This study employs a three-arm design to evaluate the effectiveness of a Home Gardening Intervention (HGI) alone and in combination with a mental health intervention (SH +). The Enhanced Usual Care (EUC) arm serves as a control, providing a benchmark for comparison while offering a higher level of support than a simple waitlist condition, thus mitigating potential nocebo effects. Participants in the EUC arm will also receive the HGI training and materials after the final 12-month data collection time point, ensuring they are not disadvantaged by participating in the control group. The HGI arm is designed to assess the impact of climate-smart agricultural practices on food security. The combined SH + HGI arm allows us to test the hypothesis that addressing mental health can enhance the effectiveness of agricultural interventions in this refugee population. This design allows for a direct comparison between the interventions and the control, as well as an assessment of the potential added value of integrating mental health support with agricultural training.

### Intervention description {11a}

Participants will be randomly assigned to one of three groups: EUC, HGI or a combined Self-Help Plus and Home Gardening Intervention (SH + HGI). In the SH + HGI group, participants will receive the Self-Help Plus intervention before starting the Home Gardening Intervention.

#### Enhanced usual care

All enrolled participants will receive EUC, which consists of a single 15-min psychoeducation session with a trained assistant overseen by a qualified mental health professional, delivered before the start of the other interventions. The session is guided by a predetermined script, addressing the issue of overthinking and offering self-management techniques [46]. Participants will also receive information on utilizing existing mental health services and UNHCR partner organizations that provide psycho-social support within the refugee settlement, as well as information about a network of trained community health workers offering basic psychosocial support.

#### Home gardening intervention

The HGI is designed to promote climate-smart farming techniques, empowering participants to achieve greater self-sufficiency [24]. The intervention provides training on growing nutrient-dense crops on small plots near participants’ homes, emphasizing year-round cultivation, sustainable farming practices and knowledge transfer [47]. The HGI utilizes a four-pronged strategy: (1) supplying tools, seeds, and other essential materials; (2) offering education on home-based sustainable agricultural practices; (3) providing lessons on nutrition and cooking; and (4) teaching sustainable harvesting and market strategies.

The program starts with a 3-day training of trainers (ToT) for 20 model farmers and the monitoring team, led by senior trainers specializing in sustainable agriculture. Village-level training of the participants includes three in-person sessions with senior trainers at model farmers’ plots, each lasting approximately 6 h, with at least 2 h devoted to hands-on practice. The timing of these sessions will be staggered across the three strata. Thereafter, trained model farmers located in each village will provide continual support to participants throughout the intervention period. Each participant will receive regular in-person visits at their home garden from a model farmer or assistant monitor.

#### Self-help plus

Self-Help Plus (SH +) is a transdiagnostic WHO intervention package based on Acceptance and Commitment Therapy that provides tools for guided self-help, empowering participants to manage stress and cope with adversity independently [39, 48]. SH + is delivered by trained, non-specialist facilitators to groups of up to 30 people via a 5-session guided self-help course. The intervention is designed to be adaptable to different cultures and languages. For this study, all SH + materials were professionally translated into Kiswahili following WHO guidelines. The Kiswahili version will be made openly available on the WHO website. The intervention teaches stress management skills through pre-recorded audio files, complemented by guided discussions among participants, using an illustrated guidebook and short audio files to practice skills outside of sessions. Two facilitators support each session by providing additional explanations, demonstrating skills alongside the audio and guiding discussions based on a course manual. The facilitators will receive specialized training from experienced mental health specialists from vivo international Uganda. These specialists will also provide ongoing supervision and support to the facilitators throughout the intervention period. This includes reviewing session plans, observing sessions in-person, providing feedback and guidance to facilitators and addressing any challenges that arise during implementation. This ensures fidelity to the SH + model and supports facilitators in delivering the intervention effectively.

### Criteria for discontinuing or modifying allocated interventions {11b}

Participants are free to withdraw from the study interventions at any time at their own request, without needing to provide a reason. In addition, the research team may discontinue or modify a participant’s allocated intervention if there are concerns about their safety or well-being. Specifically, if a participant experiences a significant clinical deterioration, including the development of severe psychiatric symptoms (e.g. psychosis, suicidality), they will be referred to specialist care within the camp. These participants may still receive the allocated intervention if deemed appropriate by the clinical team. Any deviations from the allocated intervention will be documented.

### Strategies to improve adherence to interventions {11c}

To ensure intervention fidelity and participant adherence, comprehensive training will be provided to all SH + facilitators and model farmers. For the Home Gardening Intervention (HGI), training will be based on a curriculum informed by best practices in sustainable farming and will include hands-on practice. For both interventions, the respective facilitators will undergo an intensive “Training of Trainers” (ToT) program, including theoretical instruction and practical exercises, with a focus on culturally relevant delivery while maintaining fidelity to core components. Ongoing support will be provided to both HGI model farmers and SH + facilitators. For HGI, this includes regular monitoring to support the model farmers through regular in-person visits to participants’ home gardens. For SH +, this includes ongoing supervision from experienced mental health specialists from vivo international Uganda, involving session plan reviews, in-person observation of sessions, feedback and guidance to address challenges. The use of local facilitators who have an understanding of the context and culture will further help to build trust and rapport with participants, thus promoting adherence.

Adherence to the intervention protocols will be monitored through several methods. For HGI, model farmers will complete checklists for each training session to evaluate adherence to the core components of the intervention. Assistant monitors will observe at least 20% of all sessions and complete the same checklists. For SH +, after each session, facilitators will complete a checklist on adherence to the session’s core elements and a post-session review form to document any challenges and feedback that will be reviewed during the regular supervision. SH + supervisors will observe at least 20% of all sessions and complete checklists on facilitators’ adherence to the core components of the session.

### Relevant concomitant care permitted or prohibited during the trial {11d}

Participants will be allowed to receive any usual care available in the Nakivale refugee settlement. There are no prohibitions on concomitant care.

### Provisions for post-trial care {30}

Following the completion of the 12-month follow-up assessments, participants in the EUC arm will be offered the opportunity to receive the HGI training and materials, ensuring that all participants have access to the potential benefits of the agricultural intervention. Additionally, all participants will be provided with information about existing mental health services and support organizations within the Nakivale settlement and surrounding areas. These services may include individual counselling, support groups and referral pathways to specialized care, if needed. There are no provisions for financial compensation for trial participation beyond the refreshments and small transport reimbursements provided during the study. This is in line with common practice in research conducted in humanitarian settings, where resources are often limited. Furthermore, the interventions being evaluated are considered low-risk and potentially beneficial [24, 49], and all participants will receive at least Enhanced Usual Care. Any potential harms will be mitigated as outlined in the “Adverse event reporting and harms {22}” section.

### Outcomes {12}

Trained local enumerators will collect data at three time points: baseline, 3-months post-intervention and 12-months post-intervention. These enumerators will undergo a comprehensive training program of at least 14 full days, covering the administration of all assessment tools, proper interviewing techniques, ethical research conduct and the informed consent process. Data will be captured electronically using the KoboCollect application on tablets. Participants will have the option to complete the assessments in Kiswahili, Kinyabwisha or English, according to their preference.

#### Primary outcome

The primary outcome of this study is food insecurity, which will be measured using the Food Insecurity Experience Scale (FIES) [50] at 12 months post-intervention. The FIES consists of eight questions addressing self-reported food-related behaviors and experiences associated with increasing difficulties in accessing food due to resource constraints. The FIES is unique as it is the most widely used method for evaluating food security at the household or individual level that allows for global comparability [50, 51]. The analysis metric will be the change in the FIES score from baseline to the 12-month follow-up, and the method of aggregation will be the median FIES raw score for each group.

#### Secondary outcomes

Secondary outcomes are focused on mental health and nutritional status, and include:

Mothers’ psychological distress: Measured using the Kessler Psychological Distress Scale (K6) [44] at baseline, 3-month and 12-month follow-ups. The K6 is a six-item questionnaire that assesses symptoms of anxiety and depression. This outcome is important as psychological distress can be both a consequence of food insecurity and a barrier to engaging in adaptive behaviors, such as those promoted by the HGI. The analysis metric will be the change in the K6 score from baseline to each follow-up time point. The method of aggregation will be the mean K6 score for each group. The 3-month assessment will also serve as a manipulation check to verify the effectiveness of the SH + intervention.

Household dietary diversity: Measured using the Household Dietary Diversity Score (HDDS) [52] at baseline, 3-month and 12-month follow-ups. The HDDS is a 12-item questionnaire that assesses the number of different food groups consumed by the household in the previous 24 h. Dietary diversity is a key indicator of diet quality and micronutrient adequacy, which is particularly important for vulnerable populations like refugees [53]. The analysis metric will be the change in the HDDS score from baseline to each follow-up time point. The method of aggregation will be the median HDDS score for each group.

Child malnutrition: Measured as height-for-age z-scores (HAZ) at baseline and 12-month follow-up. Chronic malnutrition, indicated by low HAZ, can have long-term consequences for children’s physical and cognitive development [54]. Height will be measured using a portable stadiometer (model: ALPHA SDM210), and weight will be measured using a digital scale with 50-g accuracy (model: Homebuds HB905). These measurements will be used to calculate HAZ scores based on the WHO Child Growth Standards, which provide age- and sex-specific reference values [55, 56]. The analysis metric will be the change in the HAZ score from baseline to the 12-month follow-up time point. The method of aggregation will be the mean HAZ score for each group.

**Fig. 1:**
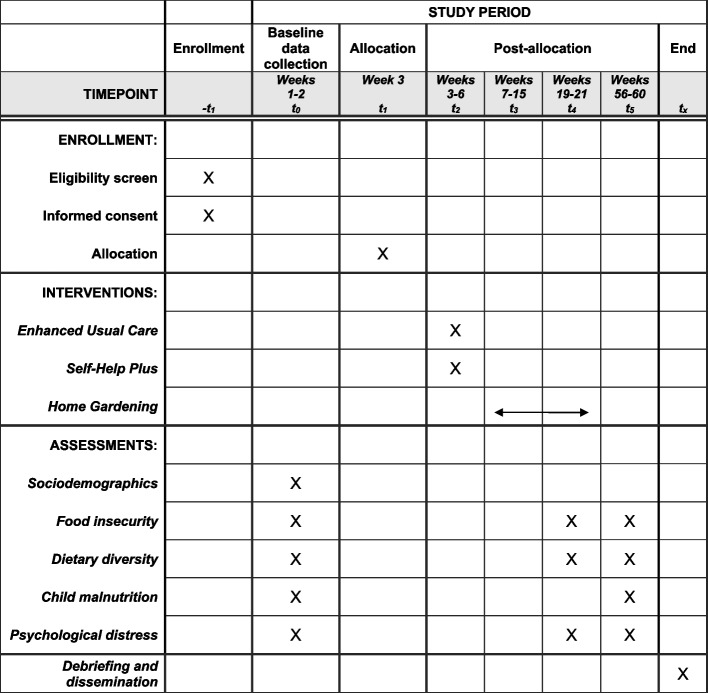
SPIRIT schedule of enrollment, interventions and assessments for the THRIVE trial

Figure 1 presents the schematic timeline of enrollment, interventions and assessments, while Table 1 provides a detailed summary of primary and secondary outcome measures.

**Table 1:** Primary and secondary outcomes of the trial

	**Instrument**	**Measure**	**Description**	**End point**	
				**3 mo.**	**12 mo.**
**Primary outcome**					
Food Insecurity	Food Insecurity Experience Scale (FIES) [50, 51]	Median of the FIES raw scores	The FIES was developed by the FAO in collaboration with Gallup and is one of the most widely used measures for tracking global food security in the context of the SDGs [50]	X	X
**Secondary outcomes**					
Maternal Psychological Distress	Kessler Psychological Distress Scale (K6) [44]	Mean score	The K-6 has been used extensively across cultural settings [44], including among South Sudanese female refugees in Uganda [49]	X	X
Household Dietary Diversity	Household Dietary Diversity Score (HDDS) [52]	Median score	The HDDS has been implemented globally in varying survey settings, including in Ethiopia and Uganda [57, 58]	X	X
Child Malnutrition	Height-for-age z-scores (HAZ) [55]	Mean z-score	HAZ is a standardized indicator of chronic malnutrition and long-term growth deficits, validated against WHO Growth Standards [55, 59]. It reliably reflects the cumulative effects of nutritional and health conditions in early childhood [55, 56, 59]		X

Figure 1 illustrates the schematic schedule for Stratum 1. Strata 2 and 3 will follow the same schedule structure, staggered with a two-week delay.

#### Exploratory outcomes

In addition to the primary and secondary outcomes detailed above, we will take advantage of the trial design to collect data on a range of other measures to explore the broader impacts of the interventions on mothers and children. These outcomes span multiple domains including economic preferences, cognitive development, social capital, parenting practices, mental health and general well-being. The results from these ancillary outcome measures will be reported in separate publications focusing on the relevant specific research areas. A full table of exploratory outcomes, including instrument details and descriptions, is available as a supplementary document (Supplementary Table S1).

### Participant timeline {13}

**Fig. 2:**
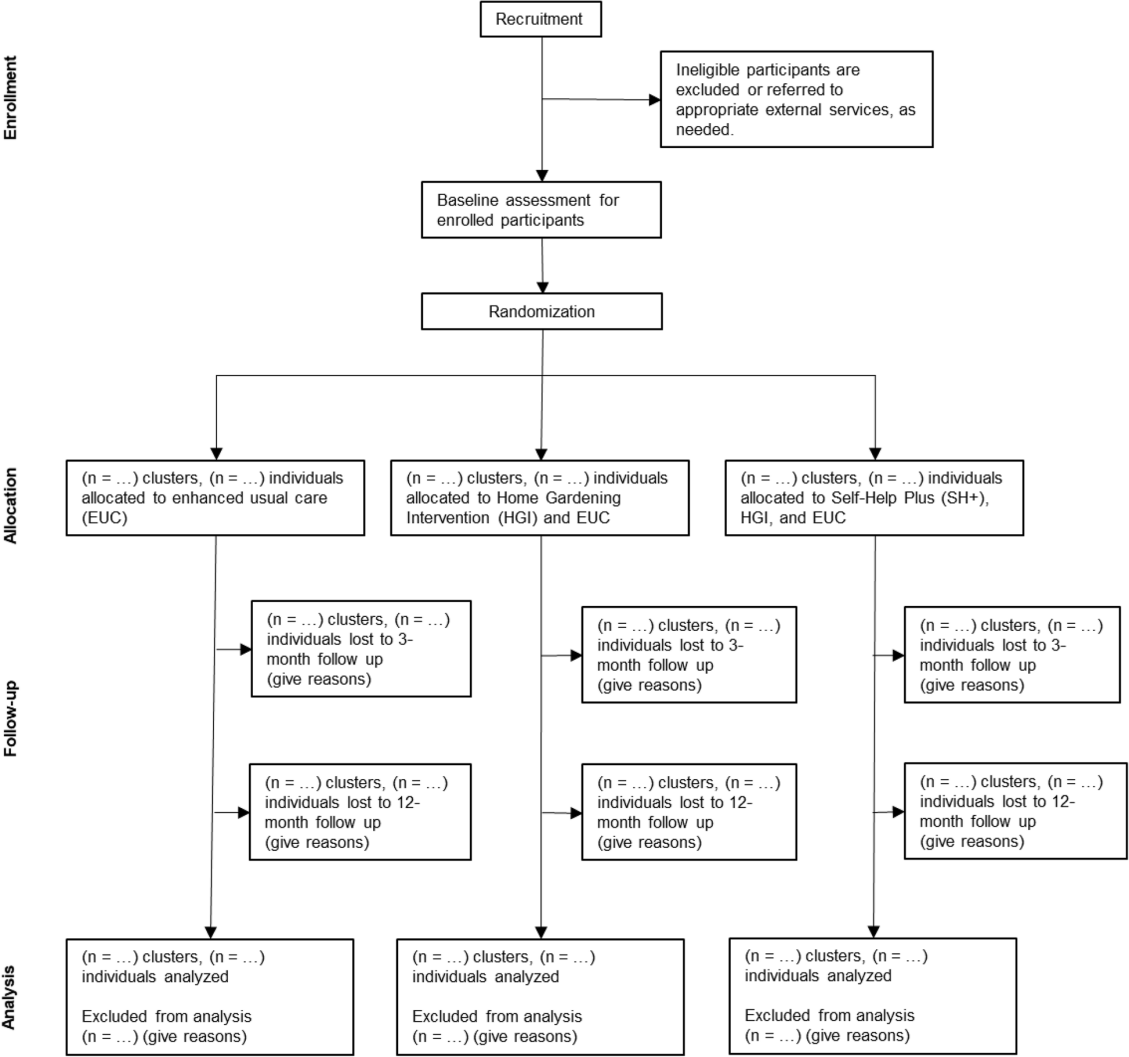
SPIRIT flow diagram of the phases of the THRIVE trial

The participant flow diagram is presented in Fig. 2. Following screening, informed consent and enrollment, baseline data collection will be conducted sequentially for each stratum: first for Stratum 1, then Stratum 2 and finally Stratum 3. Villages will be randomly assigned to one of the three study arms after the completion of baseline data collection for each respective stratum. Due to this staggered design, allocation and intervention start dates will vary across the three strata. Enhanced Usual Care (EUC) will be delivered as a single session to all participants before the start of the other interventions. The Home Gardening Intervention (HGI) involves three village-level training sessions and regular monitoring visits, the timing of which will be staggered across strata. The Self-Help Plus (SH +) intervention consists of five group sessions, also staggered across strata. Follow-up assessments will be conducted at 3 and 12 months after the intervention for each participant.

### Sample size {14}

The sample size calculation was conducted using *PowerUp* [60]*!* and was based on the primary outcome of food insecurity. A total sample size of 900 mother–child dyads (300 per arm) is required to detect a between-arm difference in the primary outcome of 0.291 standard deviations with 95% power and an alpha of 0.05. This calculation assumes an intra-cluster correlation coefficient (ICC) of 0.01, based on previous studies in similar settings [61, 62], and accounts for 50% of the variance being explained by individual-level covariates and 20% by cluster-level characteristics. The calculation also assumes a conservative 20% attrition rate at the 12-month follow-up.

### Recruitment {15}

In partnership with local agricultural experts, we will identify villages suitable for the intervention based on feasibility, focusing on those with high concentrations of new arrivals from 2021 to 2025. These individuals often experience significant psychological distress due to limited social support networks and inadequate access to basic resources. Prioritizing these villages aligns with standard livelihood intervention practices in the camp. By focusing on this group, the study aims to provide insights into how targeted support can mitigate these early vulnerabilities and improve adaptation outcomes. Key inclusion criteria for villages are sufficient access to water for home gardening and available agricultural land, while high urbanization levels and current participation in similar programs will be exclusion criteria.

We will include all villages identified in advance with the help of local experts as having concentrations of new arrivals between Q1 of 2021 and Q1 of 2024. During fieldwork, we will then ascertain with the help of local networks in the camp which of the remaining villages have concentrations of new arrivals since Q2 of 2024, and include them sequentially until we have a total of 30 villages. To reach the target number of participants in each village, contiguous villages with smaller concentrations of new arrivals will be grouped into larger units for the purposes of randomization.

Within each selected village, we will compile a list of prospective participants who meet our broad inclusion criteria, identified in conjunction with village leaders, following standard local practices used by established organizations providing livelihood programs in the camp. This list will then be randomly ordered, and individuals will be screened in this order until we reach 30 participants from each village. The random shuffling and selection process will be conducted by an experienced researcher at Bielefeld University who is not otherwise involved in the study. The date of first recruitment was December 13th, 2024.

## Assignment of interventions: allocation

### Sequence generation {16a}

Allocation will be made at the cluster level, stratified according to the settlement patterns of recent arrivals. Stratification will account for three categories based on arrival periods: (1) 2021–2022, (2) 2023 to Q1 2024 and (3) Q2 2024 until recruitment begins. Allocation will follow the Sequentially Numbered Opaque Sealed Envelopes (SNOSE) method [63].

### Concealment mechanism {16b}

The allocation sequence will be concealed using SNOSE, ensuring allocation is unknown until the moment of selection. The envelopes will contain a folded paper indicating the assigned treatment arm (EUC, HGI or SH + HGI). The principal researcher in Uganda will prepare these envelopes, which will be sequentially numbered and opaque to prevent prediction or manipulation.

### Implementation {16c}

The unmasked principal researcher in Uganda will oversee the preparation of the sealed envelopes. The SNOSE procedure will take place, one stratum at a time, in a meeting attended by village leaders or their representatives. The randomization procedure will be explained, after which each village representative will draw a sealed envelope containing the treatment assignment. The research team will not influence or intervene in the selection process. Participants within each village will then be assigned to the intervention arm allocated to their village.

## Assignment of interventions: blinding

### Who will be blinded {17a}

Due to the nature of the interventions, blinding of participants is not feasible. Data collectors will be blinded to the village allocations.

### Procedure for unblinding if needed {17b}

Unblinding will not be necessary in this trial.

## Data collection and management

### Plans for assessment and collection of outcomes {18a}

Primary and secondary outcome data along with sociodemographic characteristics and potential confounders will be collected electronically using tablets equipped with KoboCollect software. Data collection will occur at baseline, 3-month and 12-month post-intervention. Baseline and 12-month follow-up assessments will be conducted in person at a central location, while 3-month follow-up assessments will be conducted at participants'homes to minimize travel burden. Local enumerators, trained by the research team, will administer the surveys and assessments. The training will cover the administration of assessment tools, general interviewing techniques and ethical conduct of research. All measures will be available in English, Kiswahili and Kinyabwisha, depending on the preferred language of the participant. Table 1 provides a summary of the primary and secondary outcomes, including their validation status. The data collection forms are available on request. To ensure data quality, the core research team in Uppsala will conduct daily checks on the number of records uploaded and the completeness of these records.

### Plans to promote participant retention and complete follow-up {18b}

To maximize participant retention and ensure complete follow-up, several strategies will be employed. Participants will receive compensation of approximately 3 USD in local currency for each of the three survey rounds. Refreshments will be provided during training and group activities for those in the intervention arms. To reduce the burden of participation and minimize travel, 3-month follow-up surveys will be conducted in participants’ homes. Furthermore, we will maintain regular communication with participants and community leaders to foster a sense of connection and address any concerns. We have also gathered direct and close contact information and are in touch with village leaders, ensuring that we can effectively track down participants at the 12-month endpoint. Finally, enumerators will be trained to build rapport with participants and emphasize the importance of their continued involvement in the study.

### Data management {19}

Data will be entered directly onto password-protected tablets assigned to each enumerator. Data uploaded through KoboCollect will be encrypted and captured securely on the humanitarian server hosted by KoboToolBox. Enrolled participants will be assigned a unique participant ID that will be used to identify them across all three time points, facilitating linkage of data while maintaining anonymity in the dataset. The principal researcher in Uganda will be responsible for overseeing data management in the field. Data will be cleaned and checked for errors. Range checks will be performed to identify outliers.

### Confidentiality {27}

All data will be stored securely and confidentially. Participants’ identifying information will be kept separate from the survey data. Access to data will be restricted to authorized personnel only. Participants will be fully informed of their rights concerning data privacy and the use of their information, in accordance with GDPR principles.

### Plans for collection, laboratory evaluation and storage of biological specimens for genetic or molecular analysis in this trial/future use {33}

Not applicable. No biological specimens will be collected, and no genetic or molecular analyses will be conducted.

## Statistical methods

### Statistical methods for primary and secondary outcomes {20a}

For the primary analysis, the results will be based on an intention-to-treat (ITT) approach, using the initial randomization of the intervention arms. Secondary analyses will employ a per-protocol approach, including only participants who completed the allocated intervention as planned. Analyses will focus on the primary outcome of food insecurity and the secondary outcomes of household dietary diversity, child malnutrition and maternal psychological distress at the 12-month endpoint. Maternal psychological distress measured at 3 months will serve as a manipulation check to verify that SH + was effective. Analyses will be conducted at the individual level, comparing the change from baseline to follow-up between arms, adjusting for clustering and baseline score.

The preliminary analysis will compare participant sociodemographic characteristics and outcome variables across the three intervention arms at baseline. Hierarchical models will be used to examine differential effects of intervention arms on outcomes. Assuming a parametric approach, we will employ models with random effects to account for clustering at the village level and repeated measures within participants. The fixed effects included in the models will be (1) intervention, represented by two indicator variables; (2) time, measured at baseline, 3-month follow-up and 12-month follow-up; and (3) the interaction between intervention and time. Fixed effects parameters will be tested with the Wald test (*t* test, *p* < 0.05, two-sided) and 95% confidence intervals without adjustment for multiple comparisons for the global test of treatment effects. A significant interaction of intervention and time suggests differential intervention effects and will be followed by pairwise comparisons to test the primary hypotheses: the EUC group vs. the HGI group, and the HGI group vs. the SH + HGI group. Participants’ demographic characteristics, baseline levels of primary and secondary outcomes and our village stratification variable (accounting for the settlement patterns of recent arrivals) will be included as covariates in the analyses.

### Interim analyses {21b}

No interim analyses are planned.

### Methods for additional analyses (e.g. subgroup analyses) {20b}

Subgroup analyses will be conducted to explore moderation effects of initial food insecurity and psychological distress at baseline, exposure to trauma, cultural differences (tribal and national identity), pre-existing adaptive capacity (e.g. previous farming experience) and length of stay in the camp. These will be examined by including interaction terms (intervention × moderator variable).

### Methods in analysis to handle protocol non-adherence and any statistical methods to handle missing data {20c}

The primary analysis will be based on an intention-to-treat (ITT) approach. A per-protocol analysis will be conducted as a secondary analysis. Missing data will be handled using imputation estimation methods (maximum likelihood, K-Nearest Neighbors) estimation if missing at random. We will utilize a conservative last-observation-carried-forward (LOCF) approach for missing follow-up data among completers.

### Plans to give access to the full protocol, participant level-data and statistical code {31c}

The survey, anonymized replication data and statistical code will be made publicly available on the Open Science Framework (OSF) website (https://osf.io/f39t8/?view_only=12c92c69c15e455f90082aec9e11d1e).

## Oversight and monitoring

### Composition of the coordinating centre and trial steering committee {5d}

The coordinating center will be based at Uppsala University, Sweden. The coordinating center will be responsible for the overall management and coordination of the trial, including ethical approvals, data management and reporting. The trial steering committee will consist of all authors and will provide oversight for the trial. The trial steering committee will meet regularly to review the progress of the trial and address any issues that may arise.

### Composition of the data monitoring committee, its role and reporting structure {21a}

A data monitoring committee is not needed for this trial.

### Adverse event reporting and harms {22}

Adverse events will be reported to the coordinating center within 24 h of the event. The coordinating center will assess the severity of the adverse event and determine whether the event is related to the study interventions. All serious adverse events will be reported to the ethics committees.

### Frequency and plans for auditing trial conduct {23}

The trial will be audited by the Kabale University Research Ethics Committee (KAB-REC) on a regular basis to ensure that the trial is being conducted according to the protocol. The auditing process will be independent from the investigators and the sponsor.

### Plans for communicating important protocol amendments to relevant parties (e.g. trial participants, ethical committees) {25}

Any important protocol modifications (e.g. changes to eligibility criteria, outcomes, analyses) will be communicated to the relevant parties (e.g. investigators, ethics boards, trial participants, trial registries, journals, regulators).

### Dissemination plans {31a}

The results of the trial will be disseminated through peer-reviewed publications, presentations at scientific conferences and reports to relevant stakeholders. The results will also be communicated to the participants and the communities involved in the study.

## Discussion

This cRCT will evaluate the effectiveness of a climate-smart home gardening intervention (HGI), with and without an integrated mental health component (SH +), in a humanitarian setting in Uganda. This study is among the first to rigorously examine the impact of combining agricultural and mental health interventions on food security among refugees. If proven effective, the interventions tested in this study, which are designed to be scalable and sustainable, could be integrated into existing humanitarian programming in Nakivale and similar settings.

The HGI, with its focus on climate-smart agriculture, offers a potentially sustainable approach to improving food security and dietary diversity. The addition of SH +, a brief, transdiagnostic psychological intervention, addresses the critical need for mental health support in this population and may enhance the effectiveness of the HGI by improving participants’ psychological well-being.

Ultimately, this study aims to contribute to the evidence base for effective interventions that address the interconnected challenges of food insecurity and mental health among war-affected populations. The results will be disseminated widely to inform humanitarian programming and policy, with the potential to improve the lives of vulnerable populations in Nakivale and beyond. The findings will also inform efforts to adapt and scale up similar integrated interventions in other humanitarian settings globally.

## Trial status

The protocol was first registered on ClinicalTrials.gov on May 17, 2024. This manuscript refers to protocol version 3, dated January 22, 2025. Participant recruitment began on December 13, 2024, and was completed on January 22, 2025. At the time of manuscript submission, baseline data collection is ongoing. An initial attempt to publish this protocol as a registered report involved an extended review process, which delayed formal submission to Trials.

## Supplementary Information

Supplementary Material 1: Table S1. Outcomes of the THRIVE trial

## Data Availability

All data and materials will be openly available on the Open Science Framework (OSF) website at this link: https://osf.io/f39t8/?view_only=12c92c69c15e455f90082aec9e11d1e.

## Abbreviations

cRCT: Cluster-randomized controlled trial
EUC: Enhanced usual care
FIES: Food Insecurity Experience Scale
HGI: Home Gardening Intervention
HAZ: Height-for-age z-scores
HDDS: Household Dietary Diversity Score
ITT: Intention-to-treat
K6: Kessler Psychological Distress Scale
LOCF: Last-observation-carried-forward
OSF: Open Science Framework
SH+: Self-Help Plus
SNOSE: Sequentially Numbered Opaque Sealed Envelopes
