# Supplementary material for: Combining mental health and climate-smart agricultural interventions to improve food security in humanitarian settings: study protocol for the THRIVE cluster-randomized controlled trial with mothers in Nakivale refugee settlement, Uganda
Source: Trials. 2025 Sep 1;26:331. doi: 10.1186/s13063-025-09042-y (PMC12403481; doi:10.1186/s13063-025-09042-y)
Supplement: Supplementary file 1 — Supplementary Material 1: Table S1. Outcomes of the THRIVE trial [file 13063_2025_9042_MOESM1_ESM.docx]

| **Table S1.** Outcomes of the THRIVE trial | | | | | | |
| --- | --- | --- | --- | --- | --- | --- |
| **Outcome** | **Dimension** | **Measure** | **Measure Description** | **Assessment time points** | | |
|  |  |  |  | **t_0_** | **t_4_** | **t_5_** |
| Economic preferences | Patience | Investment task (1) | Participating mothers and their children will engage in a set of incentivized economic games to measure economic preferences, modeled on the approach of Chowdhury and colleagues (2). Patience will be assessed through an investment task. Children will receive one token and choose whether to exchange it immediately for sweets or invest it for two tokens—and twice the amount of candy—at the end of the assessment. Mothers will receive five tokens and decide how many to keep and how many to invest, with invested tokens doubling and paid out the following week. The number of tokens invested serves as the measure of patience | X |  | X |
|  | Social preferences | Social preference task (adapted) (3,4) | To measure social preferences, we will ask participants to make a series of binary choices between two payoff distributions. In each decision, one option always distributes rewards equally (1:1), while the other provides an asymmetric allocation. Across three games, we manipulate the asymmetric option to assess different social preferences. In the prosocial game, the alternative payoff is (1:0), measuring basic prosociality. In the envy game, the alternative payoff is (1:2), capturing the willingness to benefit the partner at a personal cost. In the sharing game, the alternative payoff is (2:0), assessing the willingness to incur a cost to benefit the partner. In the efficiency game, the alternative payoff is (2:3), where the total payoff is maximized, allowing us to measure a preference for efficiency over equality.  Both mothers and children will participate in the game, with the order of the games randomized. Each participant will play two iterations: one where the partner is described as living in their village and another where the partner is described as living in a distant village within the camp. | X |  | X |
|  | Risk | “Bomb” risk elicitation task (adapted) (5) | Risk-taking will be measured using an adapted version of the "bomb task." In this task, participants will be presented with a set of boxes—five for mothers and three for children. One of these boxes contains an illustration of a crocodile, while the remaining boxes each contain a token. Participants decide how many boxes they want to open, knowing that finding a token allows them to keep it, whereas uncovering the crocodile results in winning zero tokens for that round. The number of boxes a participant chooses to open serves as the measure of risk-taking. | X |  | X |
| Cognitive skills (children) | Mathematics ability | Free counting (6) | In the free counting task, the children are asked to count as high as they can without error. | X |  | X |
|  |  | Give-n (6) | In the Give-n task, children are asked to give a certain number of discs (e.g., Could you give the puppet three discs to play with?) from a group of discs placed in front of the child. | X |  | X |
|  |  | Number comparison (6) | In the number comparison task, children are shown a number and asked relational questions (e.g., “What comes after 4?”). | X |  | X |
|  |  | Addition/subtraction (6) | In the addition and subtraction task, children are asked to complete a sequence of both verbal and non-verbal arithmetic operations. Initially, they engage in non-verbal addition and subtraction trials, followed by verbal trials. In the addition task (e.g. 1+1), the researcher places a disc on a cardboard in full view of the child. This disc is then hidden under a cover. The researcher then slides another disc under a fabric. Next, the researcher places two discs on the other cardboard in front of the child and lifts the cover to show the two discs on the cardboard and says, "Look, yours is just like mine." This demonstration is then presented to the child again following the same procedure, but this time the child is asked to place the appropriate number of discs on the cardboard after being shown by the researcher. A verbal response is not expected. If the child has placed the wrong number of discs on the cardboard, the answer is corrected, and the researcher repeats the same procedure. The same procedure is followed as for the subtraction task (e.g., 2 - 1), but in this case the disk is removed from under the cover. In verbal addition/subtraction, the procedure remains the same; however, instead of physically placing the corresponding number of discs in front of them to represent the outcome, children are instructed to articulate the result verbally. | X |  | X |
|  | Spatial ability | 2-D TOSA (6) | In the 2D-TOSA task, children are asked to copy geometric designs, presented via a flat image on a card, using felt shapes. Performance is scored based on the accuracy of adjacent pieces, horizontal and vertical direction, and relative position of the pieces in their construction of the design. | X |  | X |
|  | Theory of mind | Surprise outcome (7) | In the surprise outcome task, children observe a story created using two dolls. One of the dolls (e.g., Sally) places a marble in her toy box. Then, Sally leaves the environment. Meanwhile, the other doll (e.g., Anne) takes the marble from Sally’s toy box and places it in her own. Anne returns and the researcher asks the child where Sally will look for her marble when Anne returns. After this false belief question, the reason for the given answer (“Why?”) is asked. | X |  | X |
|  |  | Surprise content (8) | In the surprise content task, the researcher asks the children what they believe is inside a candy box. After the child's answer, it is shown that the candy box actually contains colored pencils. After these unexpected contents are placed in the box, the children are asked what they thought was inside the box before it was opened and what their friends would think was inside the box before it was opened. After these false belief questions, the reason for the given answer ('Why?') is asked. | X |  | X |
|  | Language ability | TIFALDI (9) | In the language task, children are asked to find the picture that matches the word the researcher said (e.g., television) among four different options. The pictures are shown to the children from a booklet with four pictures on each page. | X |  | X |
| Social capital | Group membership and participation |  | Social capital will be measured using a set of survey questions assessing group membership and participation in community activities. Respondents are asked whether they have been members of various groups in the past three months, including mothers' groups, savings groups, religious groups, and political groups. Follow-up questions capture the frequency of participation in activities organized by these groups. | X |  | X |
|  | Trust |  | Trust will be measured using a series of questions assessing how much respondents trust different social groups, with responses ranging from 0 (not at all) to 4 (a lot). Trust is evaluated at multiple levels, including family, neighborhood, village, members of the respondent’s tribe in other villages, individuals from other tribes in the settlement, and people from Uganda more broadly. | X |  | X |
| Parenting | Positive parenting | 6-item Positive Parenting Subscale of the Alabama Parenting Questionnaire (APQ) (10) | The positive parenting subscale of the APQ focuses on the frequency of positive interactions between parents and children. Each item is rated on a scale from 1 (never) to 5 (always), with higher scores indicating more frequent use of positive parenting practices. | X | X | X |
|  | Child maltreatment | 11-Item Discipline Module of the Multiple Indicator Cluster Survey (MICS) (11) | MICS is a household survey developed by UNICEF. It consists of eleven Yes/No questions that inquire about the different disciplinary actions taken by the caregiver in the past month. The total score can range from 0 to 11, with higher scores indicating the use of more types of disciplinary actions. | X | X | X |
| Maternal mental health | Posttraumatic stress | Post-Traumatic Checklist 6-item Civilian Version (PCL-C) (12) | The PCL-C has 6 items rated from 1 to 5, measuring key PTSD symptoms. Total score ranges from 6 to 30, with scores above 14 indicating potential PTSD. | X | X | X |
|  | Stress | Perceived Stress Scale 4-item version (PSS-4) (13) | The PSS-4 is a brief self-report measure of perceived stress. It consists of four questions that ask about feelings and thoughts during the last month. Each item is rated on a scale from 0 (Never) to 4 (Very often), with items 2 and 3 reverse scored. The total score, ranging from 0 to 16, is obtained by adding the scores of all items. Higher scores indicate higher perceived stress. | X | X | X |
|  | Depression | Patient Health Questionnaire-9 (PHQ-9) (14) | The PHQ-9 is a widely utilized questionnaire that assesses the severity of depression in individuals. It consists of nine items, each corresponding to a symptom of depression. The responses are scored on a scale ranging from 0 (not at all) to 3 (nearly every day), with the total score indicating the depression level. The scale's range is 0-27, reflecting varying degrees of depression severity from mild to severe. | X | X | X |
|  | Anxiety | Generalized Anxiety Disorder 7-item scale (GAD-7) (15) | The GAD-7 is a brief measure designed to assess the severity of generalized anxiety disorder symptoms. It includes seven items that evaluate key symptoms such as nervousness, excessive worry, and fear. Respondents rate how often they have been bothered by each symptom over the past two weeks on a scale from 0 (not at all) to 3 (nearly every day). The total score ranges from 0 to 21, with higher scores indicating more severe anxiety. | X | X | X |
|  | Psychological flexibility | Acceptance and Action Questionnaire - version 2 (AAQ-2) (16) | The AAQ-2 consists of seven items scored on a 7-point Likert scale ranging from 1 (never true) to 7 (always true). The total score can range from 7 to 49, with higher scores indicating greater psychological inflexibility. | X | X | X |
| Maternal wellbeing and functioning | Functional impairment | 15-item version of the World Health Organization Disability Assessment Schedule 2.0 (WHODAS-II) (17) | The WHODAS-II is a standardized instrument for measuring health and disability across various domains of functioning. It covers six domains: cognition, mobility, self-care, getting along, life activities, and participation. Each item is rated for difficulty over the past 30 days on a 5-point scale from 0 (none) to 4 (extreme or cannot do). The scores can be summed up to provide a profile of functioning and disability. | X | X | X |
|  | Subjective wellbeing | WHO-5 Well-Being Index (18) | The WHO-5 is a concise self-report tool that measures an individual's subjective well-being. It contains five questions that assess positive mood, vitality, and general interests. The responses are scored on a scale from 0 (at no time) to 5 (all of the time), and the total score is then multiplied by 4 to give a final score ranging from 0 to 100. A score of 0 indicates very poor well-being, while 100 represents excellent well-being. | X | X | X |
| Child wellbeing and functioning | Child wellbeing and functioning | KINDL-R (19) | The KINDL-R is a parent-reported questionnaire of child wellbeing and functioning, consisting of 24 items across six subscales: physical well-being, psychological well-being, self-worth, family, friends, and everyday functioning (school or preschool/kindergarten). Responses are rated on a 5-point Likert scale from 1 (never) to 5 (all the time), with a total score calculated to reflect overall health-related quality of life. | X | X | X |
|  |  | Kiddy-KINDL (20) | The Kiddy-KINDL is the self-report version of the KINDL and consists of 12 items. It employs a 3-point Likert scale (never; sometimes; very often) to assess various dimensions of a child's well-being. A total score is calculated, ranging from 0 to 100, with higher scores indicating better child wellbeing and functioning. It has been validated for use in children aged 4 and above. For exploratory purposes, we will also administer it at baseline to children who are just below this age range (3>4). By the 12-month endpoint, all participating children will be within the validated age range. | X | X | X |
|  | Child emotional and behavioral problems | Pediatric Symptom Checklist (PSC-17) (21) | The PSC-17 is a brief, 17-item parent-reported screening tool for identifying emotional and behavioral problems in children aged 4-17. It includes three subscales: Internalizing (5 items, anxiety/depression), Externalizing (7 items, conduct/aggression), and Attention (5 items, impulsivity/inattention), rated on a 3-point scale (0-2). Scores range from 0 to 34, with ≥15 suggesting possible psychosocial dysfunction, and subscale cutoffs at Internalizing (≥5), Externalizing (≥7), and Attention (≥7). | X | X | X |
| The table presents the dimensions, measures, and descriptions of the exploratory outcomes, along with the time points at which assessments occur. Measures include economic preferences, cognitive skills, parenting behaviors, maternal mental health, maternal well-being and functioning, child well-being, and social capital. Assessment time points: t0 (baseline), t4 (3 months post-intervention), and t5 (endline, 12 months post-intervention). | | | | | | |

**References**

1. Angerer S, Bolvashenkova J, Glätzle-Rützler D, Lergetporer P, Sutter M. Children’s patience and school-track choices several years later: Linking experimental and field data. J Public Econ. 2023 Apr;220:104837.

2. Chowdhury S, Sutter M, Zimmermann KF. Economic Preferences across Generations and Family Clusters: A Large-Scale Experiment in a Developing Country. J Polit Econ. 2022 Sep 1;130(9):2361–410.

3. Fehr E, Bernhard H, Rockenbach B. Egalitarianism in young children. Nature. 2008 Aug;454(7208):1079–83.

4. Bauer M, Cassar A, Chytilová J, Henrich J. War’s enduring effects on the development of egalitarian motivations and in-group biases. Psychol Sci. 2014;25(1):47–57.

5. Crosetto P, Filippin A. The “bomb” risk elicitation task. J Risk Uncertain. 2013;47(1):31–65.

6. Verdine BN, Golinkoff RM, Hirsh-Pasek K, Newcombe NS. Methods for longitudinal study of preschool spatial and mathematical skills. Monogr Soc Res Child Dev. 2017 Mar;82(1):31–55.

7. Baron-Cohen S, Leslie AM, Frith U. Does the autistic child have a “theory of mind” ? Cognition. 1985 Oct;21(1):37–46.

8. Kayasili BK, Acarlar F. The Development of Theory of Mind According to False Belief Performance of Children Ages 3 to 5. Educ Sci Theory Pract. 2011;11(4):1821–6.

9. Kazak Berument S, Güven AG. Turkish Expressive and Receptive Language Test: I. Standardization, Reliability and Validity Study of the Receptive Vocabulary Sub-Scale. Turk J Psychiatry. 2013;24(3):192–201.

10. Essau CA, Sasagawa S, Frick PJ. Psychometric Properties of the Alabama Parenting Questionnaire. J Child Fam Stud. 2006 Sep 26;15(5):595–614.

11. UNICEF. MICS6 Child discipline module under 5 [Internet]. 2017. Available from: https://data.unicef.org/wp-content/uploads/2017/12/MICS6-Child-discipline-module-under-5.pdf

12. Lang AJ, Wilkins K, Roy-Byrne PP, Golinelli D, Chavira D, Sherbourne C, et al. Abbreviated PTSD Checklist (PCL) as a guide to clinical response. Gen Hosp Psychiatry. 2012;34(4):332–8.

13. Cohen S, Kamarck T, Mermelstein R. A Global Measure of Perceived Stress. J Health Soc Behav. 1983 Dec;24(4):385.

14. Kroenke K, Spitzer RL. The PHQ-9: A New Depression Diagnostic and Severity Measure. Psychiatr Ann. 2002 Sep;32(9):509–15.

15. Spitzer RL, Kroenke K, Williams JBW, Löwe B. A Brief Measure for Assessing Generalized Anxiety Disorder: The GAD-7. Arch Intern Med. 2006 May 22;166(10):1092.

16. Bond FW, Hayes SC, Baer RA, Carpenter KM, Guenole N, Orcutt HK, et al. Preliminary Psychometric Properties of the Acceptance and Action Questionnaire–II: A Revised Measure of Psychological Inflexibility and Experiential Avoidance. Behav Ther. 2011 Dec;42(4):676–88.

17. World Health Organization. World Health Organization Disability Assessment Schedule 2.0. In: The SAGE Encyclopedia of Abnormal and Clinical Psychology [Internet]. 2455 Teller Road, Thousand Oaks, California 91320: SAGE Publications, Inc.; 2017 [cited 2023 Apr 17]. Available from: https://sk.sagepub.com/reference/the-sage-encyclopedia-of-abnormal-and-clinical-psychology/i40911.xml

18. Bech P. Measuring the dimension of psychological general well-being by the WHO-5. Qual Life Newsl. 2004;15–6.

19. Ravens-Sieberer U, Bullinger M. Assessing health-related quality of life in chronically ill children with the German KINDL: first psychometric and content analytical results. Qual Life Res. 1998 Jul;7(5):399–407.

20. Villalonga-Olives E, Kiese-Himmel C, Witte C, Almansa J, Dusilova I, Hacker K, et al. Self-reported health-related quality of life in kindergarten children: psychometric properties of the Kiddy-KINDL. Public Health. 2015 Jul;129(7):889–95.

21. Gardner W, Murphy M, Childs G, Kelleher K, Sturner R. The PSC-17: a brief Pediatric Symptom Checklist with psychosocial problem subscales. A report from PROS and ASPN. Ambul Child Health. 1999;5(3):225–36.
